# Expression of long noncoding RNA *Xist* is induced by glucocorticoids

**DOI:** 10.3389/fendo.2022.1005944

**Published:** 2022-09-14

**Authors:** Yun Su, Xing Chen, Hongyan Zhou, Sean Shaw, Jie Chen, Carlos M. Isales, Jing Zhao, Xingming Shi

**Affiliations:** ^1^ Department of Neuroscience & Regenerative Medicine, Augusta University, Augusta, GA, United States; ^2^ Department of Mathematics, Logistical Engineering University, Chongqing, China; ^3^ Department of Pathology and Pathophysiology, School of Medicine, Jianghan University, Wuhan, China; ^4^ Division of Biostatistics and Data Science, Department of Population Health Sciences, Augusta University, Augusta, GA, United States; ^5^ Department of Orthopaedic Surgery, Augusta University, Augusta, GA, United States; ^6^ Institute of Interdisciplinary Complex Research, Shanghai University of Traditional Chinese Medicine, Shanghai, China

**Keywords:** glucocorticoid, *Xist*, MSC, differential expression., glucocorticoid receptor, long noncoding (lnc) RNA

## Abstract

Glucocorticoids (GCs) are potent anti-inflammatory and immunosuppressive agents. However, their clinical usage is limited by severe multisystemic side effects. Glucocorticoid induced osteoporosis results in significant morbidity and mortality but the cellular and molecular mechanisms underlying GC-induced bone loss are not clear. GC use results in decreased osteoblast differentiation with increased marrow adiposity through effects on bone marrow stem cells. GC effects are transduced through its receptor (GR). To identify novel GR regulated genes, we performed RNA sequencing (RNA-Seq) analysis comparing conditional GR knockout mouse made by crossing the floxed GR animal with the Col I promoter-Cre, versus normal floxed GR without Cre, and that testing was specific for Col I promoter active cells, such as bone marrow mesenchymal stem/osteoprogenitor cells (MSCs) and osteoblasts. Results showed 15 upregulated genes (3- to 10-fold) and 70 downregulated genes (-2.7- to -10-fold), with the long noncoding RNA X-inactive specific transcript (*Xist*) downregulated the most. The differential expression of genes measured by RNA-Seq was validated by qRT-PCR analysis of selected genes and the GC/GR signaling-dependent expression of *Xist* was further demonstrated by GC (dexamethasone) treatment of GR-deficient MSCs *in vitro* and by GC injection of C57BL/6 mice (wild-type males and females) *in vivo*. Our data revealed that the long noncoding RNA *Xist* is a GR regulated gene and its expression is induced by GC both *in vitro* and *in vivo*. To our knowledge, this is the first evidence showing that *Xist* is transcriptionally regulated by GC/GR signaling.

## Introduction

Glucocorticoids (GCs) are highly effective anti-inflammatory and immunosuppressive agents and are frequently used to treat diseases such as rheumatoid arthritis (1, 2), asthma (3, 4), and pulmonary disease (5). GCs exert their actions *via* intracellular glucocorticoid receptors (GRs) (6, 7) which, upon activation, can either directly bind to glucocorticoid response elements (GREs) on the target gene promoters and regulate their transcription (8–10), or indirectly through interaction with other transcription factors such as NF-kB and AP-1 and inhibit their transcriptional activities (11–13). It has long been known that pharmacologic GC therapy results in bone loss/osteoporosis and increases the incidence of bone fractures (14–16). However, it is also known that physiological levels of GC are required for normal bone acquisition, as demonstrated in several animal models in which GR is deleted (17) or GC signaling is disrupted in bone (18–20). Despite intensive investigations, the cellular and molecular mechanisms underlying GC actions in bone are not clear. The bone marrow mesenchymal stem cells (MSCs) are multipotent and capable of differentiating into several distinct cell lineages *in vitro*, including osteoblasts, adipocytes, chondrocytes, muscle cells, and even neuronal cells (21–24). In bone tissues, MSCs give rise to osteoblasts or marrow adipocytes governed, in large part, by marrow microenvironment and developmental stages as these two pathways are reported to have a reciprocal relationship (25–27). In this study, we aimed to identify novel endogenous genes that are regulated by GR in MSCs by analyzing GR-deficient MSCs. We found that among other significantly up- or downregulated genes in GR cKO cells, that a long noncoding RNA X-inactive specific transcript (Xist) was the most significantly affected RNA species in GR cKO cells (down by tenfold). As studies of LncRNAs in bone are sparse, we decided to focus on Xist for further validation of this RNA-Seq data and examine Xist as a potential modulator of bone turnover.

## Materials and methods

### Experimental animals

Conditional GR knockout (GR cKO) mice were generated by breeding a GR-floxed (GR^fl/fl^) mouse (28) with a 3.6 kb rat type I collagen promoter-driven Cre transgenic mouse (Col3.6-Cre) (29). Thus, the cKO mice are deficient of GR in Col I-expressing cells. Mouse genotype was confirmed by PCR analysis of tail genomic DNA using primers 5’- AATCAGAATTGCTCACTCACAA-3’ (forward) and 5’-GAACTGGAAGTAGTAACACTG-3’ (reverse). PCR analysis of Cre was performed using primers 5’-GCATTTCTGGGGATTGCTTA-3’ (forward) and 5’-GTCATCCTTAGCGCCGTAAA-3’ (reverse). C57BL/6 mice (6-month-old males and females) were obtained from the National Institute on Aging (NIA) aged rodent colonies. Mice were group housed (4 - 5 mice/cage) in the Augusta University Laboratory Animal Service facility under a 12-hr dark-light cycle and fed with standard rodent chow and water *ad libitum*. After a week of recovery, mice were intraperitoneally injected with synthetic glucocorticoid dexamethasone (Dex) at a dosage of 3 mg/kg (n = 3 mice per group) or equal volume of ethanol (vehicle control, n = 2). Twelve hours after injection, mice were sacrificed and total cellular RNAs were collected from bone tissues (femur and tibia). All animal procedures were performed in accordance with a protocol (#2008-0302) approved by the Augusta University Institutional Animal Care and Use Committee (IACUC).

### MSC isolation, cell culture and RNA isolation

Bone marrow mesenchymal stem cells (MSCs) were isolated from long bones (femur and tibia) of 6-month-old male GR cKO and GR-floxed mice (n = 3 mice per genotype) in the Stem Cell Core facility at Augusta University. The Core uses a procedure that includes a negative-immuno-depletion (using magnetic beads conjugated with anti-mouse CD11b and c, CD45R/B220, and PDCA-1) followed by a positive-immuno-selection (using anti-Sca-1 beads). The MSCs isolated using this procedure are negative for CD11b, CD11c, and CD45, and positive for Sca-1, and are capable of undergoing osteogenic, adipogenic, myogenic, and chondrogenic differentiations as demonstrated by Alizarin Red-S (ARS) staining of mineralized bone matrix, Oil Red O staining of intracellular lipid vacuoles, immunolabeling of muscle-specific proteins myosin (cytoplasmic) and myogenin (nuclear), and Alician blue staining of acidic polysaccharides (30). Importantly, they also differentiate into osteoblast-like lining cells or even incorporate into trabecular bone after injection into mice (31). Images of purified cells and data demonstrating successful deletion of GR are provided as supplementary material (**Supplementary Figure S1**). The purified MSCs were cultured under standard cell culture condition in DMEM supplemented with 10% FBS and antibiotics. Total cell lysates were collected in TRIzol reagent and sent to the Otogenetics Corporation (Atlanta, GA) for RNA isolation and RNA-Seq analysis.

MSCs used in ChIP and luciferase reporter assays were isolated from bone marrow of 6-month-old C57BL/6 mice using the same method described above.

### RNA-seq analysis

RNA QC, polyA cDNA preparation and QC, Illumina library preparation and QC were all performed at the Otogenetics Corporation. RNA-Seq were performed on a HiSeq2000 sequencing machine. A minimum of 20 million reads (2 reads x 10 million fragments) were generated per sample. Basic bioinformatics and differential expression analyses were performed on DNAnexus platform. Raw data FASTq and bioinformatic reports were delivered *via* secure Google Cloud Drive. The raw RNA-Seq data has been deposited at NCBI SRA database (ID: PRJNA862943) and the processed results provided in **Supplementary Table S1**.

### Data analysis workflow

1. The sequencing data sets from illumina HiSeq2000 (fastq.gz) were first mapped with *Tophat (v2.0.5)* against reference assembly UCSC mm9 downloaded from illumina iGenome and then, the mapped files (accepted_hits.bam) from each sample were input into *cufflinks (v2.0.2)* to locate genomic regions with expression under the guidance of ‘genes.gtf’ (annotation file from illumina iGnome). Information of expression was recorded in ‘transcript.gtf’.
2. Cufflinks.cuffmerge was called to combine all ‘transcripts.gtf’ into a single ‘merged.gtf’, and mapping files generated by Tophat were input into *cufflinks.cuffdiff (v.2.0.2)* to measure expression level on regions (genes, transcripts, CDS, etc.) defined in ‘merged.gtf’.
3. Expression levels were measured with FPKM (Fragments Per Kilobase per Million mapped), which is located in.fpkm_tracking files in cuffdiff folder. Statistical test on difference between samples is located in.diff files. The q­value less than 0.05 is considered as an indication for statistical significance.
4. Cuffdiff analysis results were implemented at different levels, including gene, isoform and CDS. All result files are tab­delimited plain text and can be opened with Excel. For definition of each field in files, please refer to manual of cufflinks at http://cufflinks.cbcb.umd.edu/manual.html.

### Construction of human gene association network

Gene association network links genes or encoded proteins by their functional interplays, including direct physical binding and indirect interaction such as their participation in a common cellular process. In this study, we used the human functional linkage network (FLN) constructed by Linhu et al. as background network (32). FLN is a densely connected and weighted network composed of 21,657 genes and 22,388,609 edges. In this network, the nodes represent genes, and edge weights the likelihood that the linked nodes participate in a common biological process. The edge weight is a probabilistic confidence score of the linkage. We normalized the original edge weight to the interval [0,1].

### Scoring network effect of a group of differentially expressed genes

A group of differentially expressed genes could affect other genes through network links. For each gene *i* in the human gene association network FLN, we quantified the influence of differentially expressed genes by a network effect score. In general, the higher score a gene receives, the deeper more pronounced it is affected by the differentially expressed genes. Specifically, a node’s *S_i_* score is defined as follows:


(3) $$S_{i}=\sum_{j=1}^{n}w_{j}^{\left(v\right)}W_{ij}^{\left(e\right)}$$

where *n* is the number of nodes in the network, $w_{j}^{\left(v\right)}$ is the weight of the node *j* defined as absolute value of log2 ratio of the expression level if the corresponding gene is differentially expressed, otherwise it is zero. $W_{ij}^{\left(e\right)}$ is the linkage weight connecting gene *i* and *j*, and it is defined as 1 when *i* = *j*.

### Real-time qRT-PCR analysis

The RNA-Seq analysis results were confirmed by real-time qRT-PCR analysis of selected genes that are differentially expressed in GR cKO and GR-floxed MSCs or the samples from bone tissues of wild type C57BL/6 mice. qRT-PCR was performed as described previously using TaqMan Reverse Transcription Reagents and a StepOnePlus Real-Time qPCR System (Thermo Fisher Scientific). The mRNA levels were normalized to β-actin and 18S rRNA (internal controls). The primer sequences used in qRT-PCR are listed below (**Table 1**). All PCR reactions were performed in triplicates and all experiments were repeated at least two times with similar results.

**Table 1:** GenBank accession numbers, primer sequences and amplicon sizes of genes used for qRT-PCR analysis.

Gene	Accession #	Forward Primer (5’-3’)	Reverse Primer (5’- 3’)	Amplicon size (bp)
*Nr3c1*	NM_008173	GGACAACCTGACTTCCTTGG	CTGGACGGAGGAGAACTCAC	108
*Xist*	NR_001463	CCTGCAAGGGATACCGTTTAT	ATGAAAGGCGAAGGAGTATGG	113
*Ldhb*	NM_001316322	CTGACCAGCGTCATCAATCA	CACAGGTCTTTGAGGTCTTTCT	104
*Aldh1a1*	NM_013467	GCAGCAGGACTCTTCACTAAA	CACTGGGCTGACAACATCATA	107
*Nsg1*	NM_010942	CCACAGGCGTAAGAACAAGA	CCAGGGAAGGAGCTAAATGAA	93
*Plac8*	NM_139198	ACTCTCTACCGAACCCGATAC	CATGGCTCTCCTCCTGTTAATG	123
*Sfrp1*	NM_013834	TGCAGTTCTTCGGCTTCTAC	CTTAGAGGCTTCCGTGGTATTG	107
*Dkk3*	NM_001360257	TCCACCGACTGCTTCAATAC	CATTCACAATCCTAGCCCTACA	108
*Gilz*	NM_010286	GGGAGTACTGACTGGTCTCTTA	CCCTCCCTCATATCGAGTCTTA	111
*18S*	NR_003278	CTGAGAAACGGCTACCACATC	GCCTCGAAAGAGTCCTGTATTG	107
*b-actin*	NM_007393	TTCTTTGCAGCTCCTTCGTT	ATGGAGGGGAATACAGCCC	149

### Western blot, immunofluorescence labeling and imaging

Western blot analysis was performed as previously described (33). In brief, whole cell lysates of GR-floxed and GR cKO BMSCs were collected in a lysis buffer. Equal amounts of total protein (40 ug) were separated on 7% SDS-PAGE, transferred onto nitrocellulose membrane, and blocked in 5% non-fat dry milk for 2 hr at RT. The membrane was then incubated with an anti-GR polyclonal antibody (1:500 dilution, Santa Cruz #sc-1002) and anti-β-actin antibody (1:1000 dilution, Abcam #ab8227) for at least 1 hr at RT. After several washes the membrane was incubated with IRDye 800 goat anti-rabbit IgG secondary antibody (1:5000 dilution, LI-COR Biotechnology) and imaged using Odyssey Infrared Imaging System.

For immunofluoresence labeling, GR-floxed and GR cKO cells were seeded in chamber slides and treated with or without 10 nM dexamethasone (Dex) for 30 min. Cells were then fixed with freshly prepared 4% paraformaldehyde containing 0.2% Triton X-100 for 15 min and blocked in 2% BSA for 1 hr at RT before incubating with anti-GR primary antibody (1:500 dilution) for at least 1 hr at RT. After several washes, the slides were incubated with goat anti-rabbit IgG-FITC secondary antibody (1:600 dilution) for 1 hr at RT in dark. The slides were washed three times in PBS for 5 min each and stained with DAPI (300 nM) to visualize the nucleus. Finally, the slides were washed, mounted with Vectorshield mounting media (Vector Laboratories), and analyzed using a Nikon TE2000 fluorescence microscope equipped with COOLSNAP Monochrome Camera. Images were acquired and processed with Metamorph Imaging System.

### Chromatin immunoprecipitation assays

ChIP assays was performed using a SimpleChIP Plus Sonication Chromatin IP Kit (#56383) and a monoclonal antibody against GR (#3660S) according to the manufacturer’s instructions (Cell Signaling Technology, Inc.). Briefly, MSCs isolated from 6-month-old male C57BL/6 mice were cultured in 150mm plates under standard cell culture condition in DMEM supplemented with 10% FBS and antibiotics. When cells reach ~90% confluency they were treated with 100 nM Dex for 30 min. to induce GR nuclear translocation. The cells were washed, cross-linked and chromatin fragmented according to the manufacturer’s instructions. The fragmented chromatin (from 2 plates) were precipitated overnight at 4°C with anti-GR or normal rabbit IgG (control). After reversal cross-link and DNA purification, the GR-bound *Xist* promoter fragments were PCR amplified with following primer pairs (numbers in brackets indicate amplicon size). GRE 1: (F) TGAAGAGCCCTTCCTTG, (R) GTAAAGGTTACTTTGTCTAACT (132bp); GRE 2/3: (F) TGTCCTTTATTATTCATGGGA, (R) GTGTCTGATCTCTTTCATGT (130bp); GRE 4: (F) GATAATTTAGGAACCAAGGA, (R) CTTCTACTTGGACAAACC (134bp).

### Transfection and luciferase assays

MSCs from 6-month-old C57BL/6 mice were transiently transfected with Xist1.2-Luc reporter construct (custom-built and sequence confirmed, VectorBuilder Inc., Chicago, IL) and the internal control pRL-null vector (Promega Corporation) using jetPEI DNA transfection reagent following manufacturer’s instructions (Genesee Scientific). 18 hr after transfection cells were treated with 10 nM of Dex for 6 hr before harvesting for luciferase activities measuement using a dual luciferase assay kit (Promega Corporation) and a Cytation 5 multifunctional reader (Agilent Technologies). Values of firefly luciferase were normalized to the renilla luciferase activity. Luciferase values shown in the figures are representative of transfection experiments performed in triplicate from three independent experiments.

### Statistical analysis

Data were analyzed by either unpaired *t*-test (Mann-Whitney) or ordinary one-way ANOVA (Tukey’s multiple comparisons test) where appropriate using Prism GraphPad software version 9.2.0. A *p*-value less than 0.05 was considered significant.

## Results

### Differentially expressed genes and the biological processes they participate in

**Figure 1:**
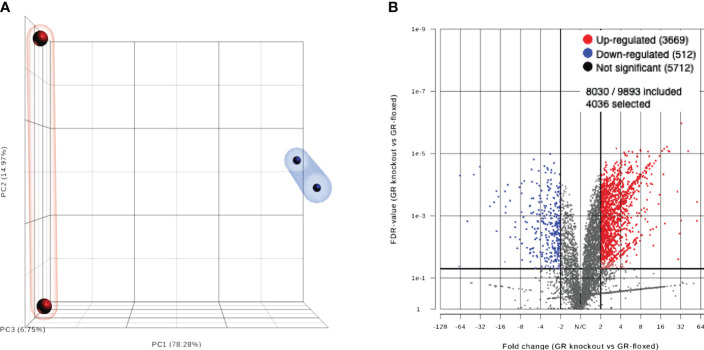
**(A)** PCA plot showing a clear separation between GR cKO and WT samples. **(B)** Volcano plot showing the number of upregulated (red dots) and downregulated (blue dots) genes. Gray dots denote no significant change.

We compared the expression levels of all genes in MSCs from GR cKO and GR-floxed (WT) mice. Principal component analysis (PCA) data showed a distinctive expression pattern between GR cKO and WT samples and a high degree of similarity between the two GR cKO samples. Though the difference between two WT samples is larger, the variation is within the limit allowed (**Figure 1A**). Statistical significance (p value) vs. magnitude of change (fold change) of genes is shown in a volcano plot (**Figure 1B**). The red and blue colors represent genes significantly up- and down-regulated, respectively, in GR cKO vs. WT cells.

**Figure 2:**
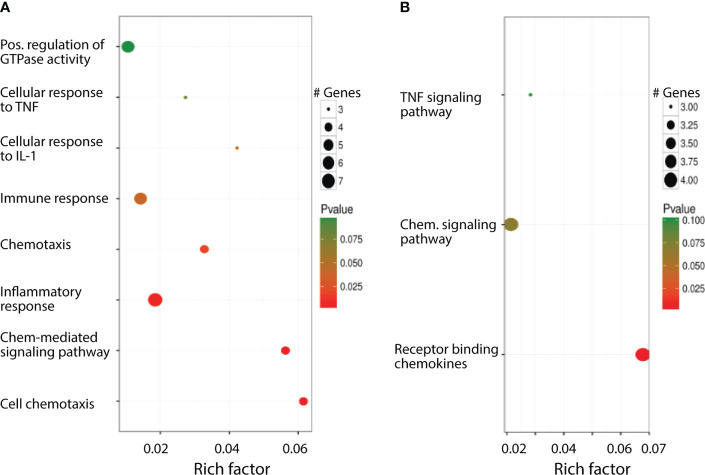
Biological processes the differentially expressed genes involved in. **(A)** Significantly enriched GO BP terms. **(B)** Significantly enriched pathways.

Normalized data set showed that 739 genes were up- or downregulated with *p* values less than 0.05. Among these 739 genes, 201 of them are unknown genes. To increase the confidence level, we analyzed only the genes whose expression in cKO vs WT has a difference larger than 3 and q-value less than 0.05 and considered these genes as differentially expressed. Results showed that 85 genes are differentially expressed, of which 78 of them have known mouse gene symbols. Since the mouse and human genes are highly conserved, we mapped the differentially expressed genes to a human data base (34) to establish potential implications of GR in human disease and health. The mapping results showed that these 85 differentially expressed mouse genes correspond to 75 distinct human genes. To explore the biological processes in which these differentially expressed genes participate, we conducted gene ontology (GO) and pathway enrichment analysis by DAVID (https://david.ncifcrf.gov/). It was found that 8 GO terms in biological process (GO BP terms) and 3 pathways, all associated with inflammation and immune response, are enriched with the identified differentially expressed genes (**Figure 2**).

### Network and pathways significantly influenced by the differentially expressed genes

**Figure 3:**
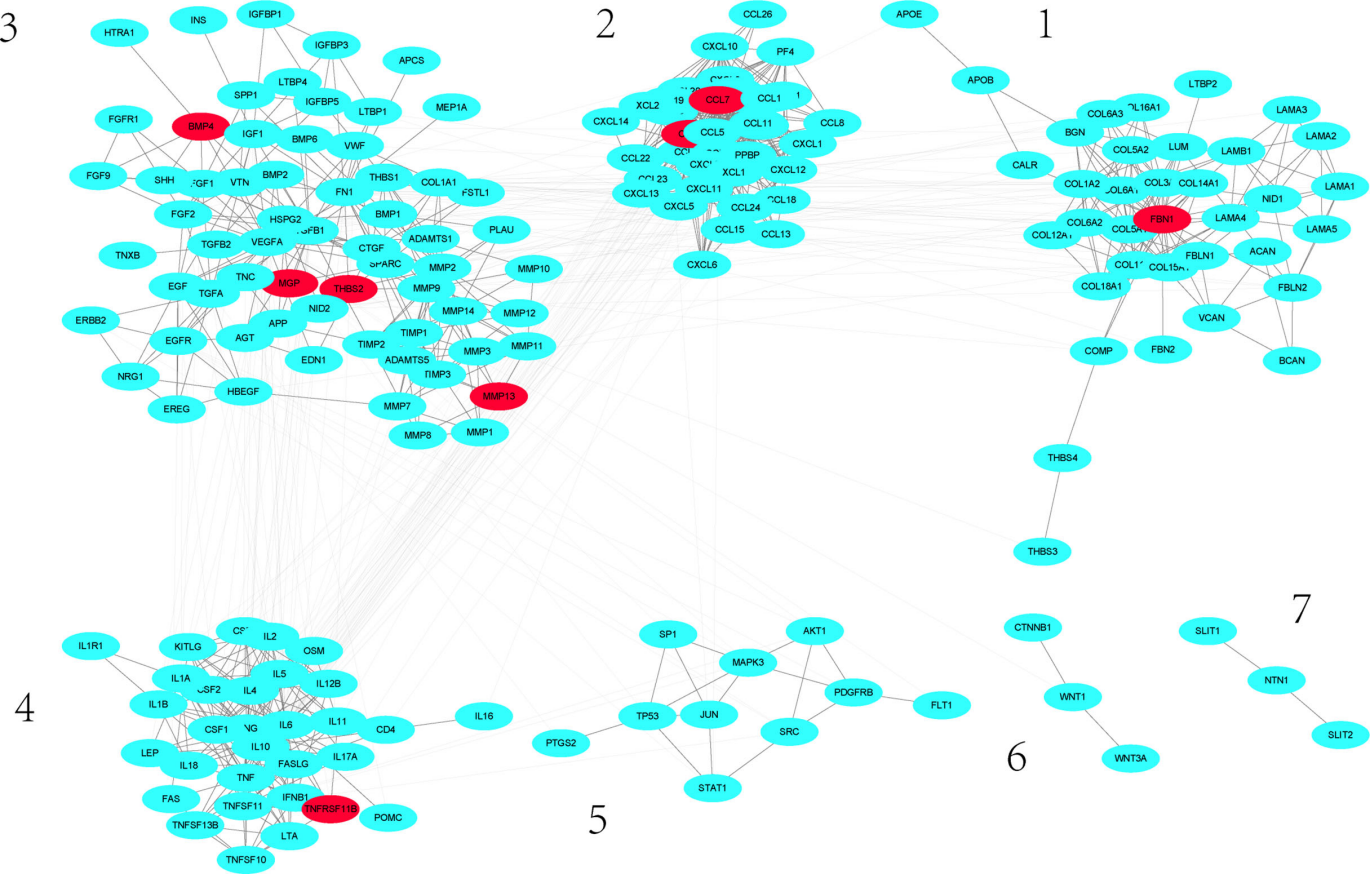
Sub-network significantly influenced by differentially expressed genes. Red nodes denote differentially expressed genes.

A group of differentially expressed genes could exert their impact on other genes through network. Using the 75 human orthologous genes as seed genes, we scored the influence of these genes to all genes in the human gene association network and took all genes whose scores were greater than 150 as significantly influenced by the differentially expressed genes. This led to 201 genes, including 11 differentially expressed genes. We took these genes and their links whose weights are larger than 0.2 from the background human gene association network. In this way, we constructed a subnetwork that is significantly impacted by the differentially expressed genes. This subnetwork includes 183 nodes and 1017 links. As shown in **Figure 3**, this subnetwork has 2 connected components. We then used simulation annealing algorithm to decompose the network into 7 dense clusters. As one can see that each cluster includes many proteins from the same families, such as CCL family in cluster 2, IL family in cluster 4, and MMP family in cluster 3, all of them are associated with inflammation.

We mapped the scores *S_i_’s* of genes to each human pathway in the KEGG pathway database and then scored pathways by the average scores of genes in that pathway. The pathways with higher scores are the ones possibly influenced by the identified differentially expressed genes. We also mapped the 201 top scored genes to the pathways to identify significantly enriched genes, and then chose the pathways that are significantly enriched with the top scored genes and also have the highest pathway scores as significantly affected by the differentially expressed genes. These pathways are summarized in **Table 2**. It can be seen that, in addition to regulating inflammation and immune response, GR cKO also affected other cellular functions including cell lineage commitment, differentiation, communication, and adhesion.

**Table 2:** Pathways significantly affected by the differentially expressed genes.

Pathway Name	Pathway Class 2	Pathway Class 1	Total Genes in Pathway	Mapped genes in pathway
ECM-receptor interaction	Signaling molecules and interaction	Environmental Information Processing	82	24
Cytokine-cytokine receptor interaction	Signaling molecules and interaction	Environmental Information Processing	273	72
IL-17 signaling pathway	Immune system	Organismal Systems	93	28
Toll-like receptor signaling pathway	Immune system	Organismal Systems	104	17
TNF signaling pathway	Signal transduction	Environmental Information Processing	108	25
Focal adhesion	Cellular community - eukaryotes	Cellular Processes	199	35
Intestinal immune network for IgA production	Immune system	Organismal Systems	49	9
Chemokine signaling pathway	Immune system	Organismal Systems	185	40
TGF-beta signaling pathway	Signal transduction	Environmental Information Processing	84	11
Hematopoietic cell lineage	Immune system	Organismal Systems	97	13
NF-kappa B signaling pathway	Signal transduction	Environmental Information Processing	95	14
Th17 cell differentiation	Immune system	Organismal Systems	107	12
Protein digestion and absorption	Digestive system	Organismal Systems	90	14
Osteoclast differentiation	Development	Organismal Systems	128	15
Th1 and Th2 cell differentiation	Immune system	Organismal Systems	92	10
T cell receptor signaling pathway	Immune system	Organismal Systems	103	11
PI3K-Akt signaling pathway	Signal transduction	Environmental Information Processing	342	46
Jak-STAT signaling pathway	Signal transduction	Environmental Information Processing	156	15
ErbB signaling pathway	Signal transduction	Environmental Information Processing	86	11
HIF-1 signaling pathway	Signal transduction	Environmental Information Processing	99	13

### Validation of the differentially expressed genes

**Figure 4:**
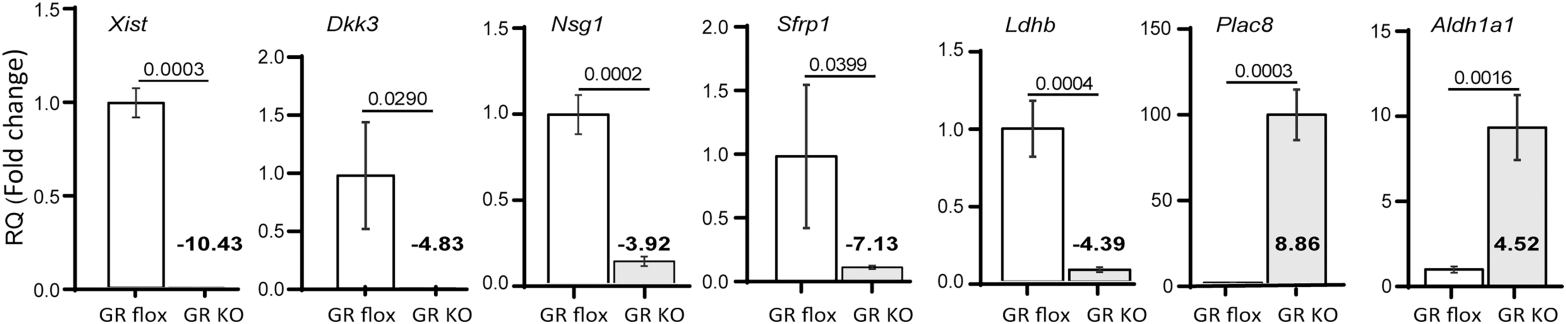
Validation of RNA-Seq data by qRT-PCR. RNA samples isolated from GR cKO and GR-floxed MSCs were analysed for the expression of Xist and indicated genes whose expression was shown to be up- or downregulated in GR cKO cells. Numbers in bold are the results from RNA-Seq analysis. qRT-PCR experiments were performed 3 times with similar results. PCR reactions were performed in triplicates. *t*-test, *p* values are indicated.

To ensure that our interpretation was based on valid RNA-Seq data, we confirmed, by real-time qRT-PCR, the differential expression of several genes shown by RNA-Seq data to be significantly up- or downregulated in GR cKO cells. These include X-inactive specific transcript (*Xist*), dickkopf-related protein 3 (*Dkk3*), neuronal vesicle trafficking associated 1 (*Nsg1*), secreted frizzled related protein 1 (*Sfrp1*), lactate dehydrogenase b (*Ldhb*), placenta specific 8 (*Plac8*), and aldehyde dehydrogenase 1 family member a1 (*Aldh1a1*). Consistent with the RNA-Seq data, qRT-PCR results showed significantly deceased mRNA levels of Xist, *Dkk3*, *Nsg1, Sfrp1, and Ldhb*, and significantly increased mRNA levels of *Plac8* and *Aldh1a1* in GR cKO cells (**Figure 4**). The *Dkk* and *Sfrp* family members are well known regulators of bone and their expression is known to be induced by glucocorticoids (35–37).

### *Xist* expression is induced by glucocorticoids

**Figure 5:**
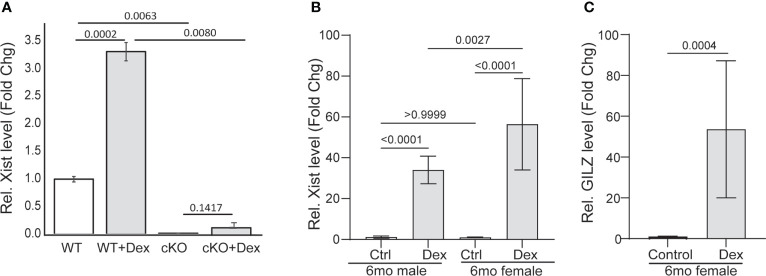
Xist RNA is induced by GCs. **(A)** qRT-PCR showing Dex induction of Xist in GR-flox and GR cKO MSCs. **(B, C)** qRT-PCR showing Dex induction of Xist **(B)** and GILZ **(C)** in mice (6-mo-old C57BL/6 mice). Results are from 2 (control) or 3 (treatment) individual mice. PCR reactions were performed in triplicates. One-way ANOVA or *t*-test, *p* values are indicated.

Ranking the significantly up- or downregulated genes of RNA-Seq data set, we found that the long noncoding RNA *Xist* was the most differentially expressed RNA species in the GR cKO cells (down by 10.4-fold). We then asked whether *Xist* expression can be induced and whether GR is required for its expression. To answer these questions, we treated GR-floxed and GR cKO MSCs with a synthetic glucocorticoid dexamethasone (Dex, 100 nM for 12 hr) and performed qRT-PCR analysis. Results showed that Dex induced *Xist* RNA expression more than 3-fold (ranging from 3 to 5 folds) in GR-floxed cells (**Figure 5A**). In contrast, *Xist* expression was not induced in GR cKO cells (**Figure 5A**). To test whether *Xist* expression is also induced by glucocorticoid *in vivo*, we intraperitoneally injected wild type C57BL/6 mice (6-month-old males and females) with Dex (3 mg/kg, n = 3) or equal volume of vehicle (ethanol, n = 2). Twelve hours after injection, mice were sacrificed and total cellular RNAs were collected from bone tissues (femur and tibia). Again, qRT-PCR results showed significant induction of *Xist* RNA in both male and female mice (>30-fold in males and >50-fold in females) (**Figure 5B**). To confirm that the increased *Xist* expression was due to the administration of glucocorticoid, we examined the level of glucocorticoid induced leucine zipper (*Gilz*), a gene known to be induced by glucocorticoids (33, 38). As expected, Dex treatment significantly increased *Gilz* mRNA expression in these samples (**Figure 5C**). It is noted that the magnitude of inductions in bone tissue was much greater than that in purified MSCs due to, most likely, the contribution from the hematopoietic lineage cells in the bone marrow. Together, these results demonstrate, for the first time, that 1) *Xist* is a glucocorticoid inducible gene, and 2) *Xist* RNA can be induced in male mice, although at a lower magnitude than in female mice.

### GR binds to *Xist* gene promoter region

**Figure 6:**
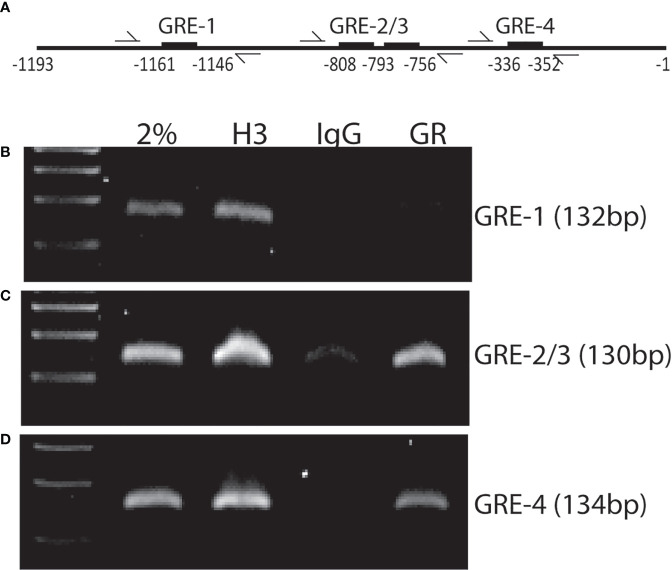
ChIP assay showing GR binding to Xist promoter region. **(A)** Schematic diagram of the approximate locations of GREs and flanking primers used in PCR reactions. **(B–D)** agarose gel images showing PCR products amplified from anti-GR antibody precipitated DNA fragments and primer pairs flanking the indicated GREs. Input: 2% sonicated DNA; H3: Anti-histone H3 mAb (positive control); IgG: normal rabbit IgG (negative control); GR: Anti-GR mAb. Experiment was performed 3 times with similar results. Shown is the results from one representative experiment.

To determine if *Xist* expression requires GR binding to the *Xist* promoter region, we performed a computational analysis using a transcription factor binding site identification software (https://tfbind.hgc.jp) and found four putative glucocorticoid response elements (GREs) within a 1.2 kb region upstream of *Xist* transcription start site (**Figure 6A**). Chromatin immunoprecipitation (ChIP) assays using wild-type MSCs and a monoclonal antibody against GR showed that, upon activation of GR with Dex (100 nM for 1 hr), GR antibodies precipitated GR-bound DNA fragments containing GRE-1 (with a lower affinity), GRE-2/3, and GRE-4 (**Figures 6B–D**). The adjacent GRE-2 and -3 are separated by only 21 nucleotides, it is not clear at this point which site GR binds to in this region. Antibody against histone H3, a universal positive control, and normal rabbit IgG served as positive and negative controls, respectively. Input DNA samples (2% sonicated DNA) was also used as a positive control for PCR reactions. Together, these results demonstrated that GR can bind to at least two out of the four putative GREs present in this 1.2 kb *Xist* gene promoter fragment.

### *Xist* promoter luciferase reporter activity

**Figure 7:**
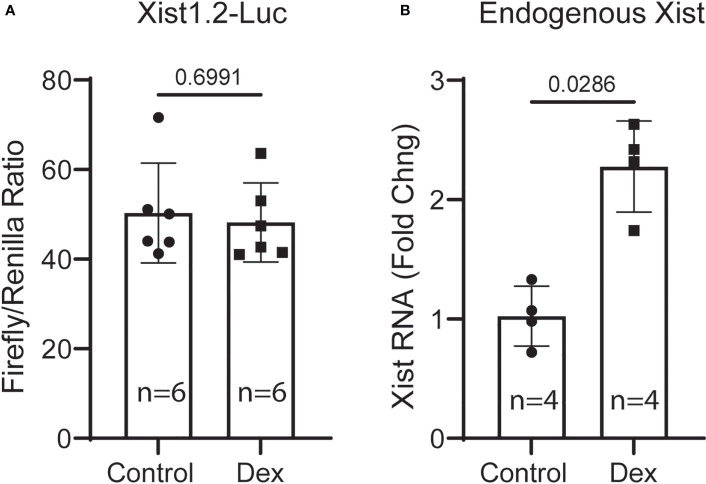
Transient transfection and luciferase-reporter assays. **(A)** MSCs were transfected with Xist1.2-Luc promoter reporter construct for 18 hr and then treated without or with 10 nM Dex for 6 hr before harvesting for luciferase activity assay. **(B)** qRT-PCR analysis of RNA samples retrieved from cell lysates **(A)** showing induction of endogenous Xist RNA by Dex in transfected cells. Experiment was performed 3 times with similar results. Shown is the result from one representative experiment performed in hextuple. RNA was retrieved from a pool of lysates (6 wells in each group) and PCR reactions were performed in quadruple. *t*-test, *p* values are indicated.

To determine the mechanism by which glucocorticoids activate *Xist* transcription, we generated a 1.2kb mouse *Xist* promoter-driven luciferase reporter construct (Xist1.2-Luc). This promoter fragment contains 4 putative GREs shown above (ChIP assay). Xist1.2-Luc plasmid, together with an internal control plasmid (pRL-null) encoding *Renilla* luciferase were co-transfected into wild-type MSCs (from 6-month-old male C57BL/6 mice) using jetPEI DNA transfection reagent (Genesee Scientific). After overnight culture (~18 hr), the transfected cells were challenged with or without 10 nM Dex for 6 hr before they were lysed. Luciferase activity was measured using a dual-luciferase reporter assay kit (Promega) and a Cytation 5 multifunctional reader (Agilent Technologies). Results showed that the Xist1.2-Luc reporter had reasonably high promoter activity (5 digits, firefly luciferase driven by Xist promoter) before normalization (to internal control, renilla luciferase expressed from promoterless vector). Unfortunately, Dex treatment showed no stimulatory effect on this promoter-reporter construct (**Figure 7A**). To confirm that the Dex reagent was biologically active and the cells were stimulated, we isolated total RNA from retrieved cell lysates used for luciferase assay and performed qRT-PCR analysis. Result showed that the endogenous *Xist* RNA was induced (**Figure 7B**), indicating that the Dex reagent was effective and this naked artificial DNA construct does not respond to GC stimulation in this setting. It is possible that long-range enhancer elements and other genes surrounding Xist locus are required for Xist expression as studies have shown that several distal enhancers are associated with *Xist*-enhancing regulatory transcript (Xert), and Xert is upregulated concomitantly with Xist and activates Xist in *cis* (39).

## Discussion

In this study, we analyzed genes that are differentially expressed in glucocorticoid receptor (GR) deficient mouse bone marrow mesenchymal stem cells (MSCs). The analysis was performed in a blind fashion using data generated from deep sequencing analysis of RNA samples prepared from purified mouse MSCs of conditional GR knockout (GR cKO) and GR-floxed control mice. The purpose of this RNA-Seq study was to identify novel genes whose expression is regulated by GR for further studies on the role of these genes in MSC differentiation and bone formation. In addition to a list of protein coding genes, some of which are known to be regulated by glucocorticoids and play important roles in bone development (35–37), our data unexpectedly revealed, that the expression of a long noncoding RNA (LncRNA), X-inactive specific transcript (*Xist*), was the most downregulated gene in GR cKO cells (tenfold lower than in GR-floxed cells). This data was confirmed by qRT-PCR analysis (**Figure 4**) and further, by glucocorticoid (Dex) treatment of GR cKO and GR-floxed cells *in vitro* as well as by Dex treatment of mice (**Figure 5**). To our knowledge, this is the first evidence showing that *Xist* is transcriptionally regulated by glucocorticoid/GR signaling. *Xist*, located on the X chromosome, was identified as a female-specific gene and functions in *cis* to silence the transcription of one of the two X chromosomes in females to regulate sex chromosome dosage compensation (40–43). The current study showed that *Xist* expression can be induced by glucocorticoids in both male and female mice (**Figure 5**) though any connection between Xist expression and glucocorticoid-induced bone loss is yet to be determined. Several recent *in vitro* studies reported the role of Xist in osteoblast differentiation but the results of these publications are contradictory; with some studies showed Xist inhibits MSC osteogenic differentiation (44–47) and others showed Xist promotes MSC osteogenic differentiation (48, 49). In addition, recent evidence also showed that Xist is overexpressed in osteosarcoma and promotes cancer cell proliferation and migration *via* mechanisms such as regulation of microRNAs (miRNAs) and mTOR and other signaling pathways (50–53). *Xist* loss- or gain-of-function studies in animal models will be required to clarify the role *Xist* plays in normal bone turnover and in glucocorticoid-induced bone loss.

## Data availability statement

The data presented in the study are deposited in the NCBI SRA repository, accession number PRJNA862943.

## Ethics statement

The animal study was reviewed and approved by Institutional Animal Care and Use Committee (IACUC) Augusta University.

## Author contributions

YS, HZ and SS contributed to cell isolation, culture and qRT-PCR analysis. XC, JC, CI, JZ, and XS contributed to data analysis, manuscript writing and editing.

## Funding

This work was supported by grants from The National Institute on Aging (R01AG046248), National Institutes of Health (NIH). Research reported in this publication was supported in part by the National Institute on Aging of the National Institutes of Health under Award Numbers R01AG046248.

## Conflict of interest

The authors declare that the research was conducted in the absence of any commercial or financial relationships that could be construed as a potential conflict of interest.

## Publisher’s note

All claims expressed in this article are solely those of the authors and do not necessarily represent those of their affiliated organizations, or those of the publisher, the editors and the reviewers. Any product that may be evaluated in this article, or claim that may be made by its manufacturer, is not guaranteed or endorsed by the publisher.

## Supplementary material

Supplementary Figure 1 Characterization of GR KO MSCs. **(A)** Bright field images showing morphology of purified MSCs. **(B)** qRT-PCR showing the absence of GR mRNA in GR KO cells. Inset: Agarose gel image showing qPCR products. **(C)** FACS analysis showing percentages of CD45, CD11b, and Sca-1 positive cell populations in purified GR-flox (top panel) and GR KO (bottom panel) MSCs. **(D)** Western blot showing the absence of GR protein in GR KO cells. **(E)** Immunostaing showing nuclear translocation of GR protein in response to dexamethason (Dex) stimulation (100nM for 30min).

## References

[1] van Everdingen AA Jacobs JW Siewertsz Van Reesema DR Bijlsma JW. Low-dose prednisone therapy for patients with early active rheumatoid arthritis: Clinical efficacy, disease-modifying properties, and side effects: A randomized, double-blind, placebo-controlled clinical trial. AnnInternMed (2002) 136(1):1–12. doi: 10.7326/0003-4819-136-1-200201010-00006 11777359

[2] Kirwan JR. Effects of long-term glucocorticoid therapy in rheumatoid arthritis. ZRheumatol (2000) 59 Suppl 2:II/85–II/9. doi: 10.1007/s003930070025 11155811

[3] Corren J Nelson H Greos LS Bensch G Goldstein M Wu J. Effective control of asthma with hydrofluoroalkane flunisolide delivered as an extrafine aerosol in asthma patients. AnnAllergy Asthma Immunol (2001) 87(5):405–11. doi: 10.1016/S1081-1206(10)62922-5 11730183

[4] Fernandes AL Faresin SM Amorim MM Fritscher CC Pereira CA Jardim JR. Inhaled budesonide for adults with mild-to-moderate asthma: A randomized placebo-controlled, double-blind clinical trial. Sao Paulo MedJ (2001) 119(5):169–74. doi: 10.1590/S1516-31802001000500004 PMC11164445 11723527

[5] Walsh LJ Wong CA Oborne J Cooper S Lewis SA Pringle M. Adverse effects of oral corticosteroids in relation to dose in patients with lung disease. Thorax (2001) 56(4):279–84. doi: 10.1136/thorax.56.4.279 PMC1746020 11254818

[6] Webster JC Cidlowski JA. Mechanisms of glucocorticoid-receptor-mediated repression of gene expression. Trends Endocrinol Metab (1999) 10(10):396–402. doi: 10.1016/S1043-2760(99)00186-1 10542396

[7] Kumar R Thompson EB. Gene regulation by the glucocorticoid receptor: Structure:Function relationship. J Steroid Biochem Mol Biol (2005) 94(5):383–94. doi: 10.1016/j.jsbmb.2004.12.046 15876404

[8] Morrison N Eisman J. Role of the negative glucocorticoid regulatory element in glucocorticoid repression of the human osteocalcin promoter. J Bone MinerRes (1993) 8(8):969–75. doi: 10.1002/jbmr.5650080810 8213259

[9] Qin W Pan J Qin Y Lee DN Bauman WA Cardozo C. Identification of functional glucocorticoid response elements in the mouse FoxO1 promoter. Biochem Biophys Res Commun (2014) 450(2):979–83. doi: 10.1016/j.bbrc.2014.06.080 24971545

[10] Petersen DD Magnuson MA Granner DK. Location and characterization of two widely separated glucocorticoid response elements in the phosphoenolpyruvate carboxykinase gene. Mol Cell Biol (1988) 8(1):96–104. doi: 10.1128/mcb.8.1.96-104.1988 3422101 PMC363086

[11] Ray A Prefontaine KE. Physical association and functional antagonism between the p65 subunit of transcription factor NF-kappa b and the glucocorticoid receptor. ProcNatlAcadSciUSA (1994) 91(2):752–6. doi: 10.1073/pnas.91.2.752 PMC43027 8290595

[12] Jonat C Rahmsdorf HJ Park KK Cato AC Gebel S Ponta H. Antitumor promotion and antiinflammation: Down-modulation of AP-1 (Fos/Jun) activity by glucocorticoid hormone. Cell (1990) 62(6):1189–204. doi: 10.1016/0092-8674(90)90395-U 2169351

[13] Schule R Rangarajan P Kliewer S Ransone LJ Bolado J Yang N. Functional antagonism between oncoprotein c-jun and the glucocorticoid receptor. Cell (1990) 62(6):1217–26. doi: 10.1016/0092-8674(90)90397-W 2169353

[14] Dequeker J Westhovens R. Low dose corticosteroid associated osteoporosis in rheumatoid arthritis and its prophylaxis and treatment: Bones of contention. JRheumatol (1995) 22(6):1013–9. 7674223

[15] Saag KG Koehnke R Caldwell JR Brasington R Burmeister LF Zimmerman B. Low dose long-term corticosteroid therapy in rheumatoid arthritis: An analysis of serious adverse events. AmJMed (1994) 96(2):115–23. doi: 10.1016/0002-9343(94)90131-7 8109596

[16] Locascio V Bonucci E Imbimbo B Ballanti P Adami S Milani S. Bone loss in response to long-term glucocorticoid therapy. Bone Miner (1990) 8(1):39–51. doi: 10.1016/0169-6009(91)90139-Q 2306553

[17] Rauch A Seitz S Baschant U Schilling AF Illing A Stride B. Glucocorticoids suppress bone formation by attenuating osteoblast differentiation *via* the monomeric glucocorticoid receptor. Cell Metab (2010) 11(6):517–31. doi: 10.1016/j.cmet.2010.05.005 20519123

[18] Sher LB Woitge HW Adams DJ Gronowicz GA Krozowski Z Harrison JR. Transgenic expression of 11:-hydroxysteroid dehydrogenase type 2 in osteoblasts reveals an anabolic role for endogenous glucocorticoids in bone. Endocrinology (2004) 145(2):922–9. doi: 10.1210/en.2003-0655 14617568

[19] Sher LB Harrison JR Adams DJ Kream BE. Impaired cortical bone acquisition and osteoblast differentiation in mice with osteoblast-targeted disruption of glucocorticoid signaling. CalcifTissue Int (2006) 79(2):118–25. doi: 10.1007/s00223-005-0297-z 16927049

[20] Yang M Trettel LB Adams DJ Harrison JR Canalis E Kream BE. Col3.6-HSD2 transgenic mice: A glucocorticoid loss-of-function model spanning early and late osteoblast differentiation. Bone (2010) 47(3):573–82. doi: 10.1016/j.bone.2010.06.002 PMC2926146 20541046

[21] Minguell JJ Erices A Conget P. Mesenchymal stem cells. ExpBiolMed(Maywood) (2001) 226(6):507–20. doi: 10.1177/153537020122600603 11395921

[22] Pittenger MF Mackay AM Beck SC Jaiswal RK Douglas R Mosca JD. Multilineage potential of adult human mesenchymal stem cells. Science (1999) 284(5411):143–7. doi: 10.1126/science.284.5411.143 10102814

[23] Prockop DJ. Marrow stromal cells as stem cells for nonhematopoietic tissues. Science (1997) 276(5309):71–4. doi: 10.1126/science.276.5309.71 9082988

[24] Kopen G Prockop D Phinney D. Marrow stromal cells migrate throughout forebrain and cerebellum, and they differentiate into astrocytes after injection into neonatal mouse brains. ProcNatl AcadSciUSA (1999) 96:10711–6. doi: 10.1073/pnas.96.19.10711 PMC17948 10485891

[25] Verma S Rajaratnam JH Denton J Hoyland JA Byers RJ. Adipocytic proportion of bone marrow is inversely related to bone formation in osteoporosis. JClinPathol (2002) 55(9):693–8. doi: 10.1136/jcp.55.9.693 PMC1769760 12195001

[26] Meunier P Aaron J Edouard C Vignon G. Osteoporosis and the replacement of cell populations of the marrow by adipose tissue. A quantitative study of 84 iliac bone biopsies. ClinOrthop (1971) 80:147–54. doi: 10.1097/00003086-197110000-00021 5133320

[27] Justesen J Stenderup K Ebbesen EN Mosekilde L Steiniche T Kassem M. Adipocyte tissue volume in bone marrow is increased with aging and in patients with osteoporosis. Biogerontology (2001) 2(3):165–71. doi: 10.1023/A:1011513223894 11708718

[28] Kim HJ Zhao H Kitaura H Bhattacharyya S Brewer JA Muglia LJ. Glucocorticoids suppress bone formation *via* the osteoclast. J Clin Invest (2006) 116(8):2152–60. doi: 10.1172/JCI28084 PMC1518793 16878176

[29] Liu F Woitge HW Braut A Kronenberg MS Lichtler AC Mina M. Expression and activity of osteoblast-targeted cre recombinase transgenes in murine skeletal tissues. Int J DevBiol (2004) 48(7):645–53. doi: 10.1387/ijdb.041816fl 15470637

[30] Zhang W Ou G Hamrick M Hill W Borke J Wenger K. Age-related changes in the osteogenic differentiation potential of mouse bone marrow stromal cells. J Bone Mineral Res (2008) 23(7):1118–28. doi: 10.1359/jbmr.080304 PMC2679384 18435580

[31] Herberg S Kondrikova G Hussein KA Periyasamy-Thandavan S Johnson MH Elsalanty ME. Total body irradiation is permissive for mesenchymal stem cell-mediated new bone formation following local transplantation. Tissue Eng Part A (2014) 20(23-24):3212–27. doi: 10.1089/ten.tea.2013.0663 PMC4259199 24914464

[32] Linghu B Snitkin ES Hu Z Xia Y DeLisi C. Genome-wide prioritization of disease genes and identification of disease-disease associations from an integrated human functional linkage network. Genome Biol (2009) 10(9):R91. doi: 10.1186/gb-2009-10-9-r91 19728866 PMC2768980

[33] Zhang W Yang N Shi XM. Regulation of mesenchymal stem cell osteogenic differentiation by glucocorticoid-induced leucine zipper (GILZ). J Biol Chem (2008) 283(8):4723–9. doi: 10.1074/jbc.M704147200 18084007

[34] Sonnhammer ELL Östlund G. InParanoid 8: Orthology analysis between 273 proteomes, mostly eukaryotic. Nucleic Acids Res (2015) 43(Database issue):D234–D9. doi: 10.1093/nar/gku1203 PMC4383983 25429972

[35] Wang F-S Lin C-L Chen Y-J Wang C-J Yang KD Huang Y-T. Secreted frizzled-related protein 1 modulates glucocorticoid attenuation of osteogenic activities and bone mass. Endocrinology (2005) 146(5):2415–23. doi: 10.1210/en.2004-1050 15677765

[36] Mak W Shao X Dunstan CR Seibel MJ Zhou H. Biphasic glucocorticoid-dependent regulation of wnt expression and its inhibitors in mature osteoblastic cells. Calcif Tissue Int (2009) 85(6):538–45. doi: 10.1007/s00223-009-9303-1 19876584

[37] Zhou H Cooper MS Seibel MJ. Endogenous glucocorticoids and bone. Bone Res (2013) 1(1):107–19. doi: 10.4248/BR201302001 PMC4472112 26273496

[38] D'Adamio F Zollo O Moraca R Ayroldi E Bruscoli S Bartoli A. A new dexamethasone-induced gene of the leucine zipper family protects T lymphocytes from TCR/CD3-activated cell death. Immunity (1997) 7(6):803–12. doi: 10.1016/S1074-7613(00)80398-2 9430225

[39] Gjaltema RAF Schwämmle T Kautz P Robson M Schöpflin R Ravid Lustig L. Distal and proximal cis-regulatory elements sense X chromosome dosage and developmental state at the xist locus. Mol Cell (2022) 82(1):190–208.e17. doi: 10.1016/j.molcel.2021.11.023 34932975

[40] Brown CJ. Equality of the sexes: Mammalian dosage compensation. Semin Reprod Med (2001) 19(2):125–32. doi: 10.1055/s-2001-15392 11480909

[41] Brockdorff N. Chromosome silencing mechanisms in X-chromosome inactivation: unknown unknowns. Development (2011) 138(23):5057–65. doi: 10.1242/dev.065276 22069184

[42] Brockdorff N Duthie SM. X Chromosome inactivation and the xist gene. Cell Mol Life sciences: CMLS (1998) 54(1):104–12. doi: 10.1007/s000180050129 PMC11147298 9487391

[43] Gayen S Maclary E Hinten M Kalantry S. Sex-specific silencing of X-linked genes by xist RNA. Proc Natl Acad Sci United States America (2016) 113(3):E309–18. doi: 10.1073/pnas.1515971113 PMC4725534 26739568

[44] Chen X Yang L Ge D Wang W Yin Z Yan J. Long non-coding RNA XIST promotes osteoporosis through inhibiting bone marrow mesenchymal stem cell differentiation. Exp Ther Med (2019) 17(1):803–11. doi: 10.3892/etm.2018.7033 PMC6307375 30651866

[45] Yu J Xiao M Ren G. Long non-coding RNA XIST promotes osteoporosis by inhibiting the differentiation of bone marrow mesenchymal stem cell by sponging miR-29b-3p that suppresses nicotinamide n-methyltransferase. Bioengineered (2021) 12(1):6057–69. doi: 10.1080/21655979.2021.1967711 PMC8806730 34486487

[46] Chen X Ma F Zhai N Gao F Cao G. Long non-coding RNA XIST inhibits osteoblast differentiation and promotes osteoporosis via Nrf2 hyperactivation by targeting CUL3. Int J Mol Med (2021) 48(1):1–6. doi: 10.3892/ijmm.2021.4970 PMC8175064 34036379

[47] Chen S Li Y Zhi S Ding Z Huang Y Wang W. lncRNA xist regulates osteoblast differentiation by sponging miR-19a-3p in aging-induced osteoporosis. Aging Dis (2020) 11(5):1058–68. doi: 10.14336/AD.2019.0724 PMC7505278 33014522

[48] Zheng C Bai C Sun Q Zhang F Yu Q Zhao X. Long noncoding RNA XIST regulates osteogenic differentiation of human bone marrow mesenchymal stem cells by targeting miR-9-5p. Mech Dev (2020) 162:103612. doi: 10.1016/j.mod.2020.103612 32389806

[49] Feng Y Wan P Yin L. Long noncoding RNA X-inactive specific transcript (XIST) promotes osteogenic differentiation of periodontal ligament stem cells by sponging MicroRNA-214-3p. Med Sci monitor: Int Med J Exp Clin Res (2020) 26:e918932. doi: 10.12659/MSM.918932 PMC7034520 32057034

[50] Li GL Wu YX Li YM Li J. High expression of long non-coding RNA XIST in osteosarcoma is associated with cell proliferation and poor prognosis. Eur Rev Med Pharmacol Sci (2017) 21(12):2829–34. 28682435

[51] Sun X Wei B Peng Z-H Fu Q-L Wang C-J Zheng J-C. Knockdown of lncRNA XIST suppresses osteosarcoma progression by inactivating AKT/mTOR signaling pathway by sponging miR-375-3p. Int J Clin Exp Pathol (2019) 12(5):1507–17. PMC6947095 31933968

[52] Liu W Long Q Zhang L Zeng D Hu B Zhang W. Long non-coding RNA X-inactive specific transcript promotes osteosarcoma metastasis *via* modulating microRNA-758/Rab16. Ann Trans Med (2021) 9(10):841. doi: 10.21037/atm-21-1032 PMC8184472 34164475

[53] Wang W Shen H Cao G Huang J. Long non-coding RNA XIST predicts poor prognosis and promotes malignant phenotypes in osteosarcoma. Oncol Lett (2019) 17(1):256–62. doi: 10.3892/ol.2018.959 PMC6313160 30655762

