# Supplementary figures and images for: Expression of long noncoding RNA Xist is induced by glucocorticoids
Source: Front Endocrinol (Lausanne). 2022 Sep 14;13:1005944. doi: 10.3389/fendo.2022.1005944 (PMC9516292; doi:10.3389/fendo.2022.1005944)

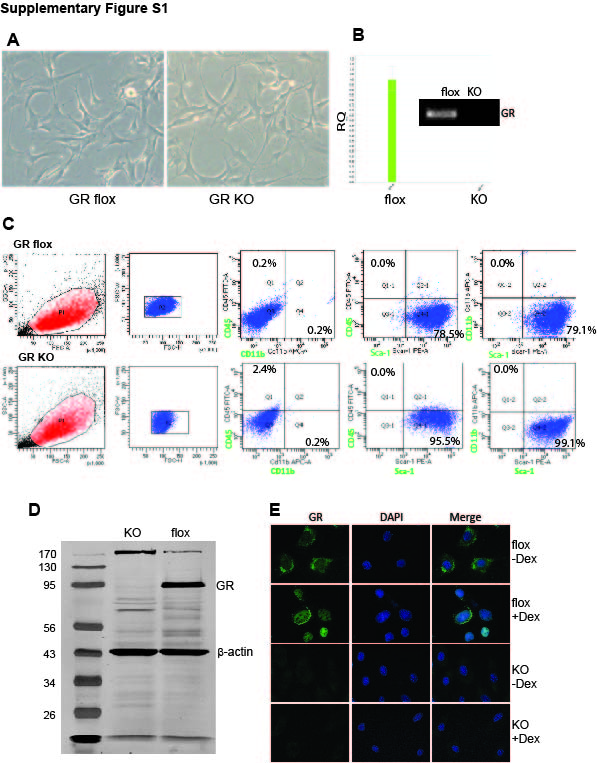

Supplement: Supplementary Figure 1 — Characterization of GR KO MSCs. (A) Bright field images showing morphology of purified MSCs. (B) qRT-PCR showing the absence of GR mRNA in GR KO cells. Inset: Agarose gel image showing qPCR products. (C) FACS analysis showing percentages of CD45, CD11b, and Sca-1 positive cell populations in purified GR-flox (top panel) and GR KO (bottom panel) MSCs. (D) Western blot showing the absence of GR protein in GR KO cells. (E) Immunostaing showing nuclear translocation of GR protein in response to dexamethason (Dex) stimulation (100nM for 30min). [file Image_1.jpg]
